# Synthesis of Triptorelin Lactate Catalyzed by Lipase in Organic Media

**DOI:** 10.3390/ijms13089971

**Published:** 2012-08-10

**Authors:** Hong Zhuang, Zhi Wang, Jiaxin Wang, Hong Zhang, Erna Xun, Ge Chen, Hong Yue, Ning Tang, Lei Wang

**Affiliations:** 1 Department of Food Science and Engineering, Jilin University, Changchun 130021, China; E-Mails: Zhuanghong@jlu.edu.cn (H.Z.); downingjlu@gmail.com (N.T.); 2 Key Laboratory for Molecular Enzymology and Engineering of Ministry of Education, College of Life Science, Jilin University, Changchun 130021, China; E-Mails: wangzhi@jlu.edu.cn (Z.W.); jiaxin1012@163.com (J.W.); Zhanghong163163@163.com(H.Z.); xunerna86@163.com (E.X.); chengeilm4196@gmail.com (G.C.); xinqingtianlan@sina.cn (H.Y.); 3 College of Chemistry, Jilin University, Changchun 130023, China

**Keywords:** triptorelin lactate, lipase, enzyme activity, esterification

## Abstract

Triptorelin lactate was successfully synthesized by porcine pancreatic lipase (PPL) in organic solvents. The effects of acyl donor, substrate ratio, organic solvent, temperature, and water activity were investigated. Under the optimum conditions, a yield of 30% for its ester could be achieved in the reaction for about 48 h.

## 1. Introduction

Peptides play important roles in the pharmaceutical and food fields [1–5]. It is generally believed that modification of a peptide can expand its scope of application. It can enhance the oral absorption of peptides and increase their enzymatic stability [6]. It can also facilitate the interaction between the peptide and its binding sites on the cell membrane, and promote a depot effect through binding to plasma proteins and the local administration site [7].

The use of enzymes for the modification of peptides has been investigated in the past decades. For example, Sih and co-workers [8–10] investigated the use of oxidative enzymes (horseradish peroxidase and chloroperoxidase) on tyrosine-containing peptides to support the synthesis of natural macrocyclic peptides. Klaus *et al*. [11] investigated the important application of carboxypeptidase Y (CPD-Y) in the modification of peptides and found that CPD-Y could catalyze the exchange of the *C*-terminal amino acid residue in peptides with various other groups (for example, conversion of peptides to peptide esters, conversion of peptides to peptide amides or conversion of peptides to other peptides). The advantages of enzymatic peptide modification are freedom from racemization, minimal activation and side-chain protection, mild reaction conditions, high regio- and stereoselectivity. Furthermore, the reactions can be carried out in a mixture of organic solvent and water, devoid of the problem of low solubility of protected peptides in the organic solvents employed in chemical synthesis.

**Scheme 1:**
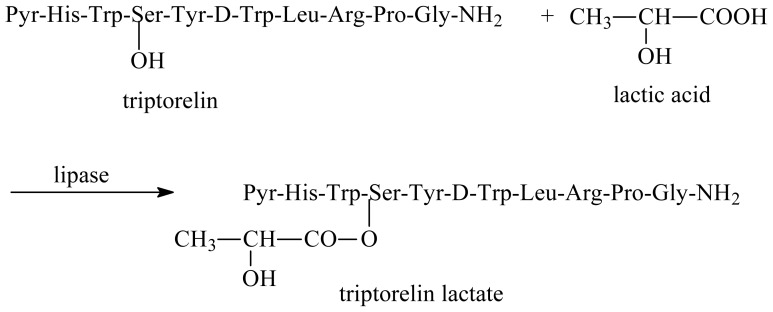
Synthesis of triptorelin lactate catalyzed by lipase.

Lipases are the most commonly used enzymes in industrial processes. They can catalyze the acylation of peptides in organic solvents. Acylation of the peptide might affect its functions, including its stability, DNA binding, protein-protein interaction, and peptide-receptor recognition. However, there still remains relatively little research so far on the applications of lipase in the modification of peptides. Herein, we report a facile lipase-catalyzed acylation of a serine-containing peptide (triptorelin) in organic media (Scheme 1), whereby the reaction conditions for the acylation have been optimized.

## 2. Results and Discussion

### 2.1. Effect of Lipase Resource

The catalytic reaction of lipase depended mainly on the type and origin of the enzyme [12]. Six commercially available lipases from various sources were selected as the catalysts for this study (Table 1). It was found that all the selected lipases could catalyze the esterification of triptorelin, but exhibited various enzyme activities. The different performances of the used lipases for esterification of triptorelin may be ascribed to their substrate specificity [13]. Because porcine pancreatic lipase (PPL) exhibited the highest activity (2.25 μmol/h/g), it was selected as the catalyst for the next study.

### 2.2. Effect of Substrate Molar Ratio

**Figure 1:**
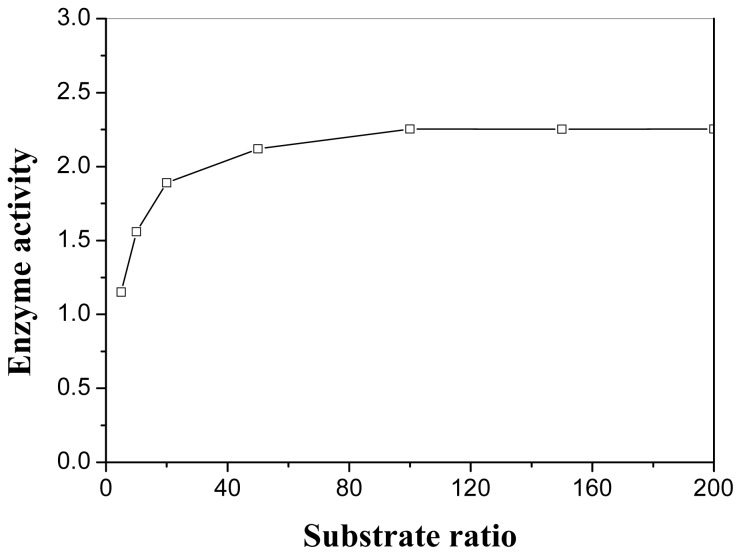
Effect of substrate ratio on the activity (μmol/h/g) of PPL in esterification of triptorelin. The reaction flask contained benzene (1.0 mL, *a*_w_ = 0.51), triptorelin (1 μmol), porcine pancreatic lipase (PPL) (5.0 mg). The reaction was performed at 40 °C and 150 rpm with different substrate ratios (lactic acid: triptorelin = 1:1–200:1).

The rate of an enzymatic catalytic reaction also depends on the concentrations of the substrate [14]. In this study, the effect of the mole ratio of lactic acid to triptorelin from 1:1 to 200:1 on esterification was investigated when the amount of the enzyme and triptorelin were kept constant (Figure 1). It was found that the enzyme activity gradually increased with increasing substrate ratio. And a ratio of 100:1 turned out to be sufficient because the enzyme activity could not be improved after this point.

### 2.3. Effect of Organic Media

Various organic solvents with different log *P* (log *P* [15], logarithm of the partition coefficient of a given solvent between *n*-octanol and water) were selected to investigate the effect of the reaction media. Log *P* is the most frequently used parameter to denote the polarity or hydrophobicity of a solvent. As shown in Table 2, the highest enzyme activity was achieved when benzene was used as reaction medium. High polar solvent may strip off the essential water from the protein and disrupt the functional structure of the enzyme, which may decrease the enzyme activity [16]. The poor solubility of triptorelin in most of the selected organic solvents may also decrease enzyme activity. Furthermore, some organic solvent molecules might alter the enzyme conformation by penetrating into the enzyme active center and then changing enzyme performance [17].

### 2.4. Effect of Temperature

**Figure 2:**
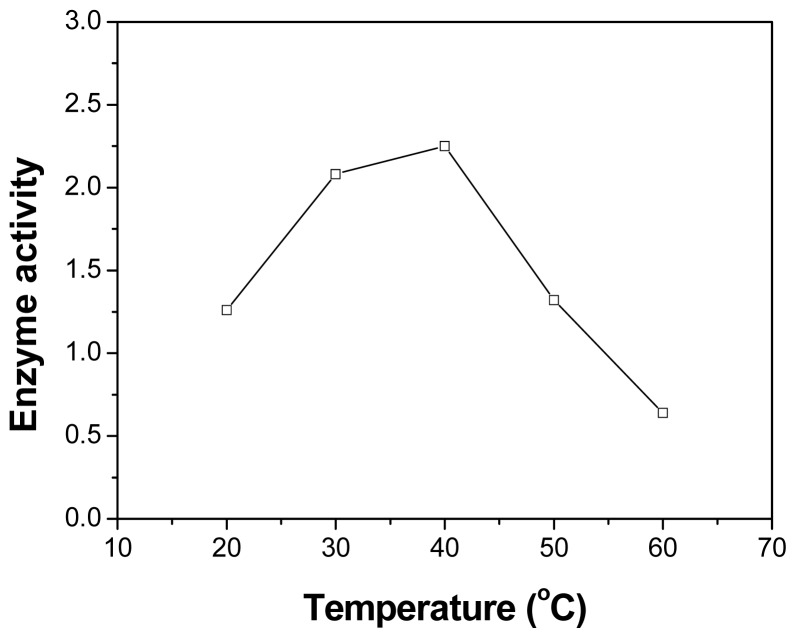
Effect of temperature on the activity (μmol/h/g) of PPL in esterification of triptorelin. The reaction flask contained benzene (1.0 mL, *a*_w_ = 0.51), triptorelin (1 μmol), lactic acid (100 μmol) and PPL (5.0 mg). The reaction was performed at various temperatures (20–60 °C) and 150 rpm.

Various temperatures were selected to examine the temperature effect on the activity of PPL in the esterification of triptorelin. As shown in Figure 2, the enzyme activity increased obviously as the reaction temperature increased from 20 °C to 40 °C. The maximal enzyme activity was obtained at 40 °C, then it decreased marginally with further increasing temperatures.

### 2.5. Effect of Initial Water Activity

**Figure 3:**
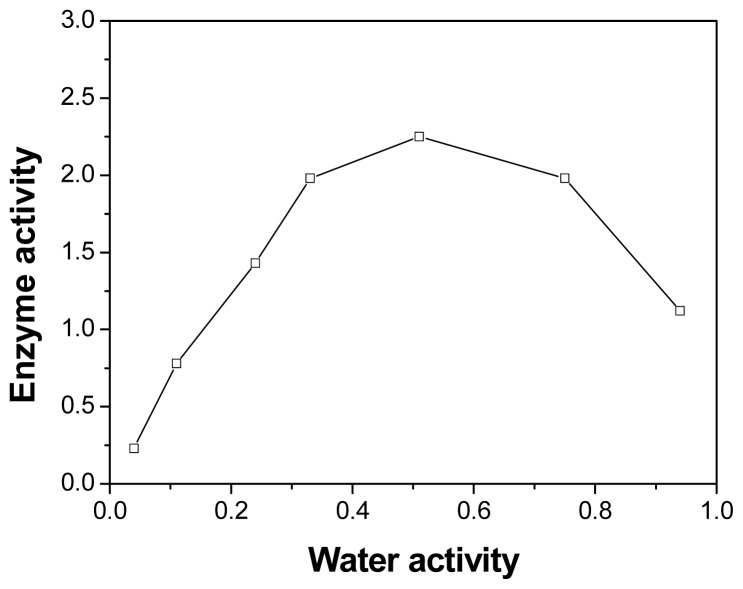
Effect of *a*_w_ on the activity (μmol/h/g) of PPL in esterification of triptorelin. The reaction flask contained benzene (1.0 mL, *a*_w_ = 0.04–0.95), triptorelin (1 μmol), lactic acid (100 μmol) and PPL (5.0 mg). The reaction was performed at 40 °C and 150 rpm.

One of the most important factors, which may affect the enzyme behavior in nonaqueous media is the water activity [18]. In the present study, the esterification catalyzed by PPL was conducted at a wide range of initial *a_w_* values (0.04–0.95). As can be observed in Figure 3, the enzyme activity exhibits a bell-shaped curve with changing water activity, and PPL exhibits the highest activity when *a_w_* = 0.51. Further decrease or increase in the initial *a_w_* value resulted in an obvious decrease in enzyme activity.

At low water activity, the conformation of PPL was excessively rigid, which might have disturbed the “induced-fit” process of PPL, and the enzyme activity was decreased [19]. At high *a*_w_ in organic solvents, the decrease in enzyme activity can be attributed to the excessively flexible conformation of lipase. The water in the reaction mixture may have acted as a competing nucleophile for the acyl-enzyme, thus suppressing the expected acyl transfer and causing an unfavorable equilibrium position in reversed hydrolysis. Hence, a high *a*_w_ had a negative effect on the thermodynamic balance, shifting the equilibrium towards hydrolysis [20].

### 2.6. Effect of the Reaction Time

**Figure 4:**
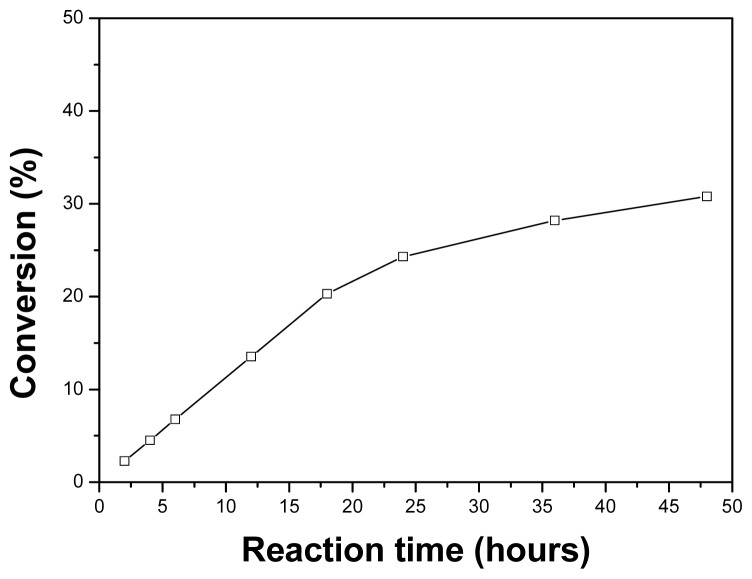
Time course of the esterification of triptorelin with lactic acid catalyzed by PPL.

It could be observed (Figure 4) that the produced triptorelin lactate increased with prolonged reaction time, and a yield of 30% for its ester could be achieved in about 48 h under optimum conditions.

## 3. Experimental Section

### 3.1. Catalysts and Chemicals

Lipase from *Pseudomonas* sp. (PSL), *Pseudomonas fluorescens* lipase (Lipase AK, AKL) and *Candida cylindracea* A.Y. lipase (AYL) was purchased from Amano Pharmaceutical Co., Ltd. (Japan). Novozym 435 was purchased from Novo (Bagsvaerd, Denmark). *Candida rugosa* lipase (CRL) was purchased from Sigma (St. Louis, MO, USA). porcine pancreatic lipase (PPL) was purchased from Shanghai Dongfeng Biochemical Reagent Co., Ltd. (China). Octreotide acetate (purity > 99%) was purchased from Shanghai TASH Biotechnology (China). Triptorelin was kindly donated by Wang Yingwu, Jilin University, China. l-lactic acid was purchased from Sigma-Aldrich Chemical Co. and was of analytical grade. Other regents were purchased from Shanghai Chemical Reagent Company (China). All the reagents and solvents were used without further purification.

### 3.2. Water Activity (a_w_) Control and Measurement

The used organic solvents were previously dried in a vacuum of 1 mmHg for 12 h. Then all the reaction mixtures with specific water activity (*a*_w_) were prepared by adding a certain amount of water. The resulting samples were pre-equilibrated at the desired temperature for 24 h in a sealed vial before being subjected to *a*_w_ measurement. The water activity (*a*_w_) was measured by Hygrolab Humidity Detector (Rotrnic, Swiss) before performing the reaction [21].

### 3.3. Esterification of Triptorelin

The reaction was performed by using triptorelin (1.3 mg, 1 μmol), lactic acid (7.5 μL, 100 μmol), benzene (1.0 mL), water activity (*a*_w_ = 0.51) and lipase (5.0 mg) while stirring at 40 °C. To determine the concentrations of the produced ester, the organic samples were withdrawn from the reaction mixture and analyzed by high performance liquid chromatography (HPLC). The synthetic activity of enzyme (μmol/h/g) was defined as the amount (in micromoles) of triptorelin ester produced per hour per gram of enzyme. Enzyme activity was determined while the conversion was controlled in the range of the 15%–30%. All experiments were repeated three times and the errors did not exceed 5%. All graphs were based on the average values.

### 3.4. Analytical Methods

The concentration of the remaining peptide and the produced ester was analyzed by HPLC. After the completion of the reaction, the products were analyzed by a HPLC system (Agilent 1100), which consisted of a binary pump, an autosampler, an oven, and a Kromasil-C18 column (5 μm, 150 × 4.6 mm i.d.). Chromatography was performed at 25 °C with a 20 min linear gradient elution from 10% to 50% acetonitrile in water containing 1% TFA at a flow rate of 1.5 mL/min. Octreotide acetate (purity > 99%) was used as internal standard. The products were detected at 280 nm. Baseline separation of triptorelin and its lactate (11.7 and 12.0 min, respectively) was obtained. Conversions were determined based on the peptide ester increase.

We used an Agilent 1100-LC system (Agilent Technologies) coupled to a 6510-ESI TOF MS (Agilent Technologies). The molecular weight and amino acid sequence of triptorelin lactate were determined by electrospray ionization mass spectrometry (ESI-MS). The *y* and *b* series of fragment ions of triptorelin lactate were obtained by in-source collision-induced dissociation (CID) and the results were shown in Table 3. The *m*/*z* of triptorelin lactate was 1383.6 (M + H)^+^ and the structure of triptorelin lactate was confirmed from the results of CID.

## 4. Conclusions

In conclusion, we describe here an effective method for the acylation of triptorelin by esterification in nonaqueous media, and PPL showed a higher catalytic performance. Under optimum conditions, the triptorelin lactate was successfully synthesized by catalyzation by PPL in benzene at 40 °C and *a*_w_ = 0.51, while lactic acid was used as acyl donor (the optimum molar ratio of lactic acid/triptorelin was 100:1). However, the activity was not satisfactory even under optimum conditions. Some techniques (for example, co-solvents, ultrasound and microwave irradiation) are currently in the process of being studied to further improve the properties of the enzyme and will be reported on in due course.

## Figures and Tables

**Table 1:** Effect of lipase resource on the esterification of triptorelin *.

Lipase	Porcine pancreatic lipase (PPL)	*Pseudomonas* sp. (PSL)	*Pseudomonas fluorescens* lipase (Lipase AK, AKL)	*Candida cylindracea* A.Y. lipase (AYL)	*Candida rugosa* lipase (CRL)	Novozyme 435
Activity (μmol/h/g)	2.25	0.67	1.32	0.54	0.27	1.47

* The reactions were carried out under the following conditions: benzene (1.0 mL, *a*_w_ = 0.51), triptorelin (1 μmol), lactic acid (100 μmol) and lipase (5.0 mg) at 40 °C.

**Table 2:** Effect of organic solvents on the esterification of triptorelin catalyzed by PPL*.

Organic solvents	Log *P*	Enzyme activity (μmol/h/g)
Dichloromethane	1.25	1.75
Benzene	2.0	2.25
Toluene	2.5	1.95
Cyclohexane	3.2	1.87
*n*-hexane	3.5	1.45
*n*-Heptane	4.0	0.88
Isooctane	4.5	0.33

* The reactions were carried out under the following conditions: different organic solvents (1.0 mL, *a*_w_ = 0.51), with triptorelin (1 μmol), PPL (5.0 mg) and lactic acid (100 μmol) at 40 °C.

**Table 3:** *b* and *y* series ions of triptorelin lactate.

Fragment ions	Structure	Calculated *m*/*z*	Measured *m*/*z*
b1	Pyr	112.04	-
b2	Pyr-His	249.10	249.1
b3	Pyr-His-Trp	435.18	435.1
b4	Pyr-His-Trp-Ser(lactate)	594.23	594.2
b5	Pyr-His-Trp-Ser(lactate)-Tyr	757.29	-
b6	Pyr-His-Trp-Ser(lactate)-Tyr-d-Trp	943.37	943.4
b7	Pyr-His-Trp-Ser(lactate)-Tyr-d-Trp-Leu	1056.46	-
b8	Pyr-His-Trp-Ser(lactate)-Tyr-d-Trp-Leu-Arg	1212.56	1212.6
b9	Pyr-His-Trp-Ser(lactate)-Tyr-d-Trp-Leu-Arg-Pro	1309.61	-
(M+H)^+^	Pyr-His-Trp-Ser(lactate)-Tyr-d-Trp-Leu-Arg-Pro-Gly-NH_2_	1383.66	1383.6
y9	His-Trp-Ser(lactate)-Tyr-d-Trp-Leu-Arg-Pro-Gly-NH_2_	1272.63	-
y8	Trp-Ser(lactate)-Tyr-d-Trp-Leu-Arg-Pro-Gly-NH_2_	1135.57	1135.4
y7	Ser(lactate)-Tyr-d-Trp-Leu-Arg-Pro-Gly-NH_2_	949.49	949.3
y6	Tyr-D-Trp-Leu-Arg-Pro-Gly-NH_2_	790.44	790.3
y5	D-Trp-Leu-Arg-Pro-Gly-NH_2_	627.37	-
y4	Leu-Arg-Pro-Gly-NH_2_	441.29	441.2
y3	Arg-Pro-Gly-NH_2_	328.21	328.1
y2	Pro- Gly-NH_2_	172.11	172.1
y1	Gly-NH_2_	75.03	-
